# Ferroptosis spreading through propagative signals

**DOI:** 10.70401/EXO.2026.0011

**Published:** 2026-05-26

**Authors:** Saloni K. Hombalkar, Jyotirekha Das, Michael Overholtzer

**Affiliations:** 1 Cell Biology Program, Memorial Sloan Kettering Cancer Center, New York, NY 10065, USA; 2 BCMB Graduate Program, Weill Cornell Medical College, New York, NY 10065, USA; 3 Gerstner Sloan Kettering Graduate School of Biomedical Sciences, Memorial Sloan Kettering Cancer Center, New York, NY 10065, USA

**Keywords:** Ferroptosis, propagation, lipid peroxidation, iron, lysosome, glutathione peroxidase 4, ferritin, erastin

## Abstract

Ferroptosis is a regulated mechanism of cell death caused by the uncontrolled peroxidation of cellular lipids that has an unusual ability to propagate or spread between cells. Here, we review studies identifying regulators of ferroptosis propagation to consider a working model that might explain this unusual feature. Recent findings implicating the spread of lipid peroxides and iron suggest that these main catalysts of ferroptosis may also be primary vehicles that can spread death between cells. Experiments revealing localized versus long-range effects of propagation are discussed, as well as sensitizing factors that may expand the propagative potential of death induced by Class II ferroptosis-inducing compounds. As ferroptosis propagation may underlie the loss of large groups of cells in degenerative diseases and may also have a specialized role in normal development, consideration of how ferroptosis can spread through propagative signals may be important for understanding both normal tissue dynamics and disease.

## 1. Introduction

Cell death is typically balanced with proliferation in normal tissues but becomes unbalanced in disease. Too much cell death, or too little, is a main driver of the development of degenerative conditions or cancer^[1]^. Cell death rates can also be rapidly adjusted in normal tissues to facilitate large-scale turnover^[2,3]^, and to shape developing tissues that are first generated with supernumerary cells and are then sculpted by cell death^[4,5]^. Among many different types of cell death that have been discovered, only a few have been shown to function in normal tissue homeostasis or development^[6]^. Many others are observed in pathological conditions and can target cells that are dysfunctional or infected^[7]^. This review will focus on ferroptosis^[8]^, an iron-dependent cell death mechanism that kills cells through the toxic accumulation of oxidized membrane lipids^[9,10]^. Ferroptosis occurs in degenerative diseases and pathological tissue damage^[11]^, and its induction is considered a promising strategy for the treatment of cancer^[12]^. Connections to normal physiology are also just emerging, with potential specialized functions mediating large-scale tissue sculpting during development^[13]^.

Ferroptosis was first discovered through its induction by chemical compounds called ras-selective lethal 3 (RSL3) and erastin^[8]^. Erastin treatment reduces levels of the key antioxidant molecule glutathione in cells by inhibiting the system x_c_- (solute carrier family 7 member 11 (SLC7A11)) amino acid antiporter, through which cystine, a rate-limiting precursor of glutathione synthesis, is taken into cells^[8,14,15]^. RSL3 targets glutathione peroxidase 4 (GPX4), an enzyme that uses glutathione as a co-factor to reduce lipid peroxides^[15,16]^. Erastin and RSL3 are examples of a now expanded set of ferroptosis inducers (FINs) that are categorized as either “Class I” compounds that deplete glutathione, or “Class II” compounds that directly inhibit GPX4.

At its core, ferroptosis involves oxidation reactions that target bis-allelic carbons of polyunsaturated fatty acids (PUFAs) in membrane lipids, ultimately leading to the formation of lipid peroxides. If lipid peroxides are not detoxified, they can spread by chain reactions within membranes, inducing damage that leads to cell death^[17]^. Ferroptosis generally requires the presence of iron to support Fenton-like reactions that convert lipid hydroperoxides to hydroxyl radicals that drive lipid peroxidation, although enzymatic functions mediated by iron-binding proteins can also play a role^[8,18]^.

While numerous cell-autonomous regulators of ferroptosis are now described, reviewed extensively elsewhere^[19]^, less understood is an unusual property of this form of cell death to spread non-cell-autonomously, or to propagate through cell populations by killing cells *in trans*^[20]^. While such “bystander” effects have been reported with other forms of death such as apoptosis, the ability of ferroptosis to spread is more unusual, manifesting as wave-like phenomena that can synchronously, and completely, eliminate large cell populations^[20–22]^. Propagative activity may explain the large-scale necrotic deaths that can target whole cell populations in degenerative conditions, for example kidney tubules in acute kidney injury and chronic kidney disease^[23,24]^, or groups of neurons that are progressively damaged and lost in degenerative diseases such as Parkinson’s disease and amyotrophic lateral sclerosis (ALS), conditions where ferroptosis is implicated^[25,26]^. Ferroptosis propagation was also recently shown to regulate normal tissue morphogenesis during development of the avian limb, where synchronous waves of death were observed to sculpt developing muscle tissue, physically separating distinct muscle groups that control the movement of different limb segments^[13]^. How ferroptosis propagation is regulated to achieve such population-scale deaths had remained obscure, but now numerous studies have revealed key factors that can control this non-cell-autonomous activity, discussed comprehensively in a recent review^[27]^. Here, we will discuss different model systems that have revealed propagative activity to consider a working model of how ferroptosis might spread through cell populations.

## 2. Evidence for Ferroptosis Propagation.

Ferroptosis propagation was first identified due to peculiar patterns of cell death that were evident in live cell imaging studies. Renal tubules treated *ex vivo* with erastin exhibited synchronized death responses that could spread between cells and were inhibited by treatment with the lipophilic antioxidant and ferroptosis inhibitor ferrostatin-1, leading the authors to posit that the observations *“suggest a direct cell-to-cell communication to deliver the deadly signal”*^[24]^. Similarly, cultured cells treated with specialized nanoparticles that deliver iron exhibited synchronous death patterns that could spread and eliminate large groups of cells through a domino-like effect^[28]^. Such “waves of death” were sensitized by starvation of cells for amino acids or treatment with erastin, and were shown to be ferroptotic by inhibition with the iron chelator deferoxamine (DFO) and antioxidants including ferrostatin-1^[28]^. Since these initial observations, propagative activity has been reported in many additional contexts involving different cell types and modes of ferroptosis induction. Some of the recently studied cell systems that have revealed propagative activity include: (*i*) a co-culture model where mouse embryo fibroblasts (MEFs) treated with erastin or the GPX4 inhibitor RSL3 were shown to cause the death of untreated cells *in trans*^[29]^; (*ii*) a cell injection model where the introduction of truncated oxidized phospholipids into HT1080 fibrosarcoma cells was shown to induce the ferroptotic death of neighboring un-injected cells^[30]^; (*iii*) a co-culture system where reintroduction of the gene expression regulator *Bach1* into immortalized knockout MEFs caused the death of non-transduced cells^[31]^; (*iv*) an optogenetics method where the turnover of GPX4 within individual HeLa cervical carcinoma cells induced the death of untreated neighboring bystander cells^[32]^; and (*v*) a blue light-based illumination system that, when combined with erastin, caused ferroptotic death waves in retinal pigment epithelial cells that could spread and eliminate large regions of unilluminated cells^[13]^. The commonality of these observations suggests that death propagation is a core feature of ferroptosis that is likely to be executed through a shared mechanism.

## 3. Localized Versus Long-Range Propagation

**Figure 1.**
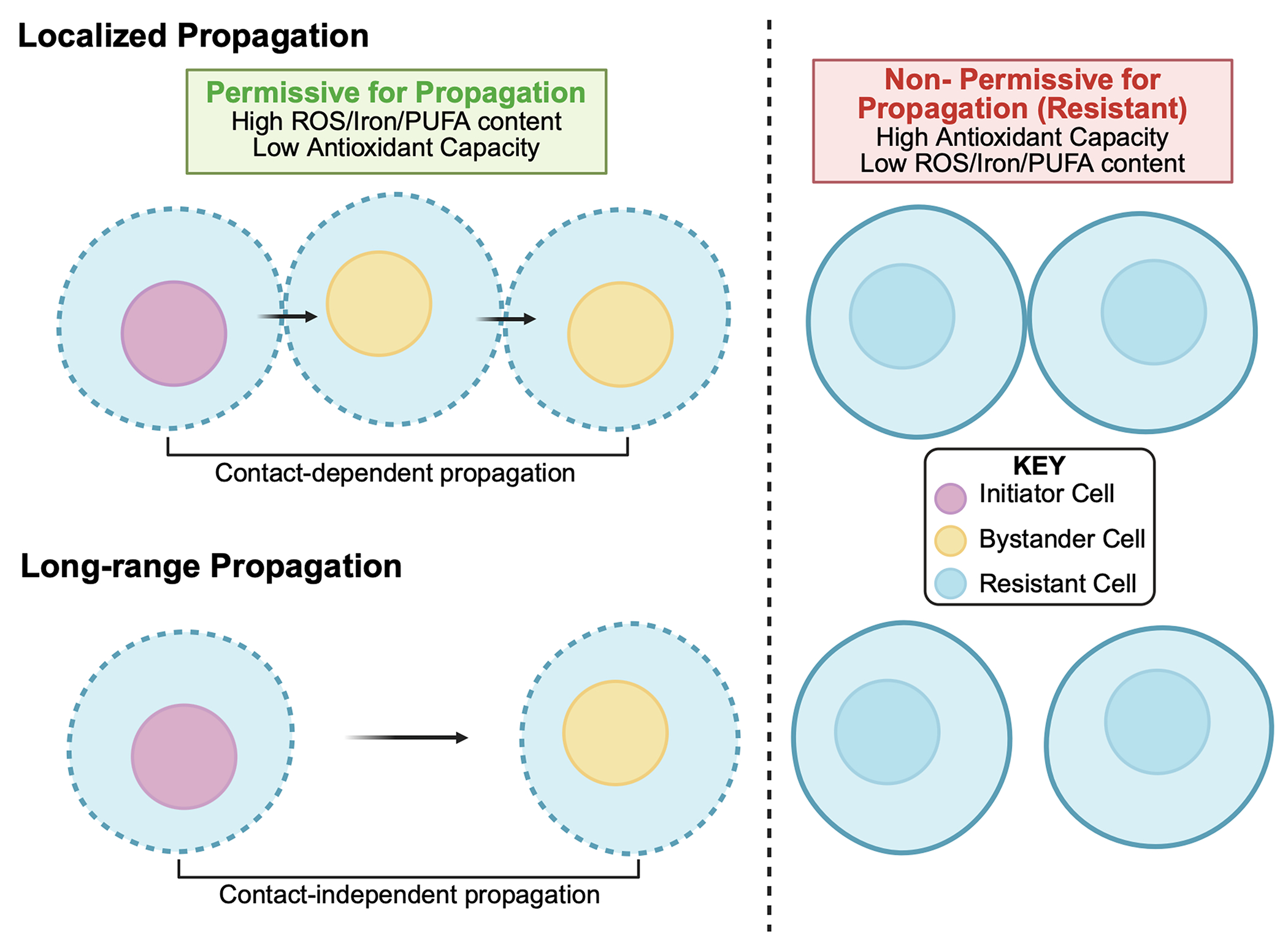
Different distance-dependent effects of ferroptosis propagation. Top: localized propagation spreads from initiator cells to direct neighboring bystander cells in a manner that requires cell-cell adhesions. Such “contact-dependent propagation” may proceed stepwise from initiator to bystander cells, provided that cells are permissive to receive and amplify incoming propagative signals. Right cells show possible bystander cells that are non-permissive for propagation, which would terminate cell death waves. Potential non-permissive states could include high antioxidant capacity, low reactive oxygen species or iron, and low PUFA content of membranes. Bottom: long-range propagation can transmit ferroptotic death in a manner that does not require cell-cell adhesions and can spread between cells spaced at least 100 microns apart. Such “contact-independent propagation” may also require bystander cells that are in a permissive state. Long-range propagation may also involve sensitization of bystander cells to death at increasing distances. Right cells show similar non-permissive states as in top row. PUFA: polyunsaturated fatty acid; ROS: reactive oxygen species.

The propagative activity of ferroptosis has been shown to have different distance-dependent effects in different cell systems that warrant consideration (Figure 1). First, propagation has been shown to occur in response to the degradation of GPX4 that can spread only locally, or from treated initiator cells to direct neighboring bystanders with shared cell-cell adhesions^[32]^. Death can then spread beyond first neighbors to additional bystander cells through a domino-like effect. Propagation in this case was shown to require direct contact between neighboring cells mediated by adherens junctions, in a manner where the junctional protein α-catenin was required, and N- or P-cadherin-mediated junctions were permissive, but E-cadherin-mediated junctions were inhibitory for death spreading. Rates of propagation in this system were also shown to be enhanced by modulating cell adhesions through manipulation of myosin II contraction.

Longer-range effects of propagation, on the other hand, have also been shown that can lead to waves of death that synchronously eliminate large groups of cells in a manner not requiring cell-cell adhesions^[13,33]^. Such death waves exhibit significant non-random spatiotemporal patterns when quantified through a computational metric called Spatial Propagation Index, or SPI^[33]^, and can jump over gaps of considerable distance, more than 100 microns, clearly demonstrating long-range propagative activity^[13]^. Like localized propagation, long-range propagation has been shown to occur through a domino-like effect, spreading at a constant speed between cells over large distances^[13,33]^. This behavior is consistent with regulation by “trigger waves” that involve amplification within individual cells of signals that are sent and received *in trans*.

While treatment of cells with Class I FINs that deplete glutathione, such as erastin, consistently induce long-range propagation, intriguingly direct inhibitors of GPX4, or Class II FINs, do not^[13,33]^. Recently it was shown that death induced by Class II FINs also results in heterogeneous death fates, where clusters of cells are observed to die with apoptotic rather than necrotic features. Apoptotic deaths in this case were also shown to lack the propagative activity of necrotic deaths^[34]^. Heterogeneous responses to GPX4 inhibition could further be converted into homogeneous necrosis with long-range propagative activity by first starving cells for amino acids or depleting glutathione, conditions that mimic pre-treatment with Class I FINs^[34]^. So, as glutathione depletion is sufficient to shift the propagative activity of Class II death responses from localized to long-range, a likely model emerges that the spreading of death over large distances may require sensitization of recipient cells to death-inducing factors. Lowering the threshold of antioxidant capacity through glutathione depletion, for example, would sensitize cells to oxidative signals sent *in trans*, enabling the propagating wave to spread over greater distances, until limited by the intrinsic range of death-inducing factors. Such a model is perhaps consistent with the induction of light-induced ferroptotic waves that could jump over large gaps when cells were also pre-treated with erastin, as well as the observed spreading of reactive oxygen species (ROS) to cells spaced farther apart than death can propagate^[13]^, a scenario in which even sensitized cells may be able to buffer against diminishing levels of death-inducing signals that are received at larger distances.

## 4. Factors That Could Control Ferroptosis Propagation

### 4.1 Lipid peroxides

Numerous studies have shown that ferroptosis propagation can be inhibited by treatment with lipophilic antioxidants such as ferrostatin-1 and liproxstatin-1, which scavenge lipid hydroperoxyl radicals^[13,24,28–30,33]^. Liproxstatin-1 can block propagation even after large-scale death waves have been initiated, suggesting that peroxidized lipids are one spreadable factor that can control propagation^[33]^. As lipid peroxidation is known to propagate through the plane of individual membranes, spreading by peroxyl radical chain reactions that progressively oxidize the unsaturated acyl chains of adjacent phospholipids, a likely, and perhaps simplest model for death propagation could involve the same chemical reactions spreading peroxidation directly between adjacent cells. A recent study indeed showed that lipid peroxidation, measured by oxidation of the lipophilic fluorescent probe C11-BODIPY, could spread from cells induced to undergo ferroptosis by optogenetic degradation of GPX4 to untreated neighboring cells^[32]^. Reporters of lipid peroxidation and cellular ROS also mark the wave fronts of large-scale propagations and can transfer between cells when imaged in other systems^[13,31]^. Neighboring cell death during localized propagation can be further enhanced by plating cells on unsaturated phospholipid substrates, a condition that facilitates the spreading of lipid peroxides, a clear sign that the direct spreading of lipid peroxidation within membranes is one vehicle that can promote the propagation of cell death^[32]^.

So, if lipid peroxides are a vehicle that can drive propagation, what is their cellular source? For localized propagation that requires cell-cell adhesions, lipid peroxides could spread directly between adjacent cells, in a manner perhaps similar to the direct spread of lipid peroxides between closely apposed giant unilamellar vesicles (GUVs) *in vitro*^[32]^. But while the direct transfer of lipids through cell-cell contacts could be possible^[35]^, it may also be unlikely^[36]^, in which case cell junctions could facilitate propagation by first trapping lipid peroxides that are released from damaged membranes and into the intercellular space. Peroxidation is known to induce numerous structural changes to lipids, including bending and “whisker” formation, where fatty acyl chains access the aqueous environment, as well as truncation at the *sn-2* position^[37–39]^. These alterations significantly deform lipid bilayers, leading to membrane thinning and increased curvature, increased rates of lipid flip-flop, pore formation, and micellization^[33,39]^. Micelles can merge into and disrupt lipid bilayers, particularly when they are damaged^[40,41]^, and they can also be readily endocytosed, potentially spreading lipid peroxides from sender cells into the lysosomes of recipients through endocytosis.

Pore formation, on the other hand, is known to lead to the influx of calcium and to generate osmotic imbalances that lead to cell rupture^[33,42,43]^. Intriguingly, localized propagation was shown to require the presence of extracellular calcium, but in a manner that supported cell-cell adhesions rather than directly regulating bystander cell death^[32]^. Porous membranes can also recruit repair factors such as endosomal sorting complex required for transport (ESCRT) proteins^[44]^, shown to localize to the plasma membrane during ferroptosis^[42]^, which could contribute to generating extracellular vesicles from dying cells that would also spread lipid peroxides^[45,46]^. In this case, spreading could again occur by direct membrane fusion of vesicles, or by their uptake through endocytosis, an activity that would deliver lipid peroxides into the lysosomes of recipient cells.

In addition to the plasma membrane, intracellular membranes could also contribute as sources of lipid peroxides. Lysosomes, which have been implicated in ferroptosis^[47–49]^, were recently linked to the execution of ferroptosis with a high degree of propagative activity^[34]^. Lysosomes accumulate lipid peroxides on their limiting membranes in response to treatment with FINs^[47]^, causing activation of the lysosome biogenesis transcription factor EB (TFEB)^[50]^, a hallmark of lysosome stress, and resulting, ultimately, in high rates of lysosome rupture^[34]^. The inhibition of cathepsin enzymes that are normally contained within lysosomes delays the execution of ferroptotic death^[34,48]^, suggesting that lysosome damage resulting from lipid peroxidation leads to rupture and the release of lysosomal enzymes that regulate necrosis. But how might lysosomes be involved in spreading lipid peroxides between cells? Lysosomes are known to become multivesicular in response to stress^[51,52]^, forming intraluminal vesicles from their limiting membranes that can ultimately be released from cells as exosomes. Lysosome exocytosis is further known to be upregulated by increases in cytosolic calcium resulting from plasma membrane damage^[53,54]^, conditions that mimic pore formation during ferroptosis induction, suggesting, altogether, a plausible scenario where lysosomal vesicles harboring lipid peroxides could be generated and released from ferroptotic cells^[53,54]^. Exosomes, like vesicles generated from the plasma membrane, would be endocytosed and delivered to the lysosomes of bystanders, potentially destabilizing their membranes^[55]^. This model is consistent with the observed high rates of lysosome rupture that occur sequentially within dying cells as ferroptosis propagates stepwise through cell populations^[34]^.

### 4.2 Iron

Iron has been consistently implicated, alongside lipid peroxides, not only as a catalyst for ferroptosis induction but also as a spreadable factor that could be mobilized between cells. Iron chelators, like lipophilic antioxidants, can inhibit ferroptosis propagation even when added to cells after large-scale death waves have been initiated^[13,33]^. Liproxstatin-1, an inhibitor of propagation and lipophilic antioxidant, may also target iron, curiously within lysosomes, as a major part of its inhibitory mechanism^[47]^. Partial iron chelation can also slow, while iron supplementation can accelerate, the speed of propagating waves, and increased levels of intracellular iron have been observed at wave fronts and shown to transfer between cells^[13,31]^. Localized propagation induced by optogenetic GPX4 degradation has further been shown to require the presence of extracellular iron, not for the initial deaths of sender cells, but to support the death of cells that are killed *in trans*, a result that implicates iron as a critical sensitizing factor to support ferroptosis propagation^[32]^. It is possible, then, that iron, like lipid peroxides, could spread directly between cells and promote the propagative activity of ferroptosis.

**Figure 2.**
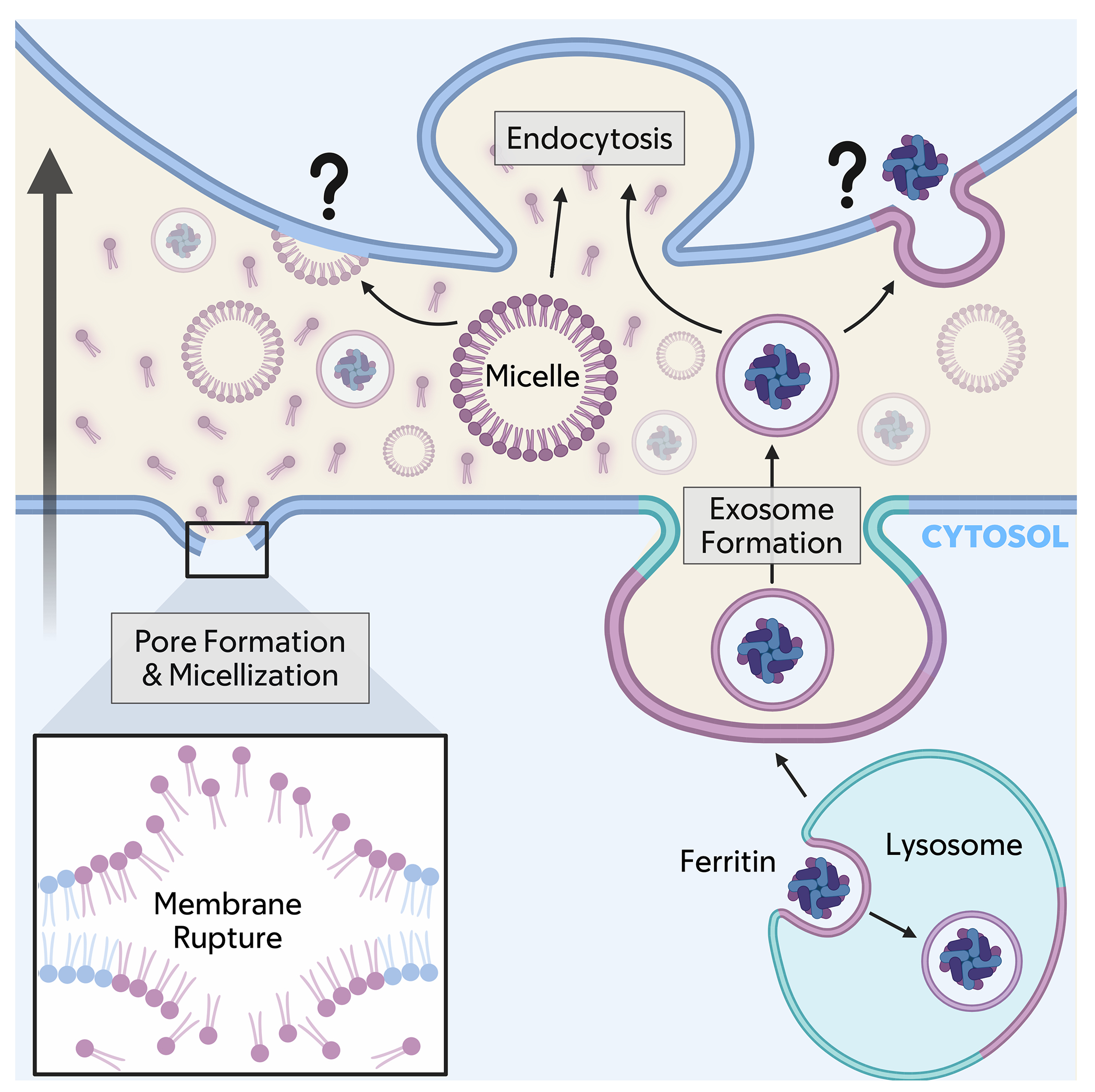
Working model for ferroptosis propagation. Lipid peroxides (shown in purple), and potentially iron, could spread from a dying, or sender cell (bottom) to a neighboring, or recipient cell (top; direction of propagation shown with large black arrow) through membrane sources derived from the plasma membrane (micelles are shown and vesicles are also possible) or lysosomes through exocytosis (an exosome releasing lipid peroxides and ferritin is shown). Membrane carriers are likely taken into neighboring cells through endocytosis, but fusion with the plasma membrane is also possible. Fragmented lipid species could also be released from sender cells and taken into recipients by endocytosis. Additional factors that may contribute to propagation including secreted proteins and RNAs are discussed in the text.

While labile iron can be exported from cells through ferroportin into the intracellular space^[56]^, potentially enabling the spread of Fe^2+^ between cells, recent studies have also shown that intracellular Fe^3+^-containing ferritin complexes can be packaged into exosomes and secreted from cells following treatment with GPX4 inhibitors or erastin. This process involves increased generation of multivesicular bodies and is dependent on upregulation of transmembrane proteins CD63^[57]^ or prominin2^[58]^. Exosome-mediated secretion of ferritin opposes lysosomal ferritin turnover, thereby limiting the generation of free iron pools within cells that are known to promote ferroptosis induction. Accordingly, prominin2-dependent secretion drives ferroptosis resistance^[58]^. However, the uptake of exosomes by neighboring cells through endocytosis would be predicted to deliver ferritin to their lysosomes, where its turnover would upregulate labile iron pools and increase ferroptosis sensitivity, as lysosomal iron in particular is increasingly linked to ferroptosis execution^[47,59]^. Thus, the exosome-dependent transfer of ferritin complexes could have two different, opposing effects that impact propagation: one that delays ferroptosis induction within sender cells, and another that sensitizes recipients to ferroptosis by upregulating labile iron pools that are predicted to promote lipid peroxidation. Through this mechanism, cells could spread *in trans* the two primary inducers of ferroptosis through lysosome-derived exosomes: lipid peroxides, and also iron (Figure 2).

### 4.3 Other secreted factors

Acting in addition to these propagative catalysts could be other kinds of signals, such as lipid fragments^[30]^ or secreted proteins^[60,61]^, that modulate recipient cell responses to incoming death inducers, potentially adjusting the relative likelihood that trigger waves might be propagated. For example, the release of *sn*-2 truncated oxidized phospholipids from damaged membranes, called platelet-activating factor (PAF) and platelet-activating factor-like phospholipids (PAF-LPLs), has been shown to function in ferroptosis propagation in kidney tubules through a mechanism involving endocytic uptake in bystander cells^[30]^. These end products of lipid peroxidation are signaling molecules but can also destabilize membranes in a signaling-independent manner, and may be deposited into the lysosomes of bystanders following endocytosis where they could significantly disrupt membrane integrity^[30]^. The secretion of a specialized β-galactoside binding protein, called Galectin-13 (Gal13) from initiator cells has also been shown to contribute to propagation through a mechanism involving downregulation of the cell surface expression of SLC7A11 on bystanders. Gal13 secretion is induced in dying cells by the lipid peroxidation-dependent activation of Protein Kinase C beta II (PKCβII), which phosphorylates and activates the forkhead box K1 (FOXK1) transcription factor that induces Gal13 expression. Secreted Gal13 then binds the cell surface receptor CD44 on bystander cells and displaces its binding to SLC7A11, leading to SLC7A11 endocytosis and downregulation, ultimately reducing glutathione synthesis in cells adjacent to those that are dying. These findings demonstrate two different sensitizing mechanisms that could regulate propagation by lowering the threshold of resistance to death in bystanders, a conclusion supported by the observed sensitization of cancer cells to ferroptosis induction by treatment with Gal13 protein or a CD44-binding Gal13 mimetic peptide^[60]^, and resistance to ferroptosis promoted by treatment with exogenous PAF-acetylhydrolase (II) (PAFAH2), a phospholipase that targets PAF and PAF-LPLs^[30]^.

Additional factors that have been shown to regulate propagation include mitochondria, which have been shown to transfer between cells in numerous contexts unrelated to ferroptosis^[62–64]^, and have been linked to the induction of ferroptosis when transferred from macrophages to different recipient cell types including cardiomyocytes^[65]^ and pancreatic beta cells^[66]^. Their uptake can affect mitochondrial networks and initiate metabolic and gene expression changes that can sensitize to ferroptosis induction. Non-coding RNAs as well have been shown to transfer through small extracellular vesicles and play a role in mediating synchronous ferroptosis observed in kidney tubules, and they can regulate the expression of numerous factors that influence lipid composition and redox potential^[27,67–69]^. These findings may underscore complex influences over *in trans* spreading of death through the control of gene expression^[70]^, a model suggested as well by transcriptional profiling^[71]^ and regulation of the induction of propagative ferroptosis by the transcription factor BACH1^[31,72]^.

## 5. Conclusion and Perspectives

Here, we have considered a working model that may explain the unusual ability of ferroptosis to propagate and spread through cell populations in wave-like patterns, drawn from both data-driven conclusions and hypothesis-generating speculation. One potential, and perhaps simplest model, could involve the dispersal of lipid peroxides, and possibly iron, the two main catalysts of ferroptosis, from dying cells, referred to as initiators, to their neighboring bystanders. The spread of these factors could initiate chain reactions of lipid peroxidation, known to propagate within the planes of individual membranes, between adjacent cells. The continuous handover of death from one cell to another would then involve amplification and resending of these incoming signals through trigger waves, characterized by intracellular activation of ROS-driven feed-forward reactions that promote lipid peroxidation^[13]^. Likely vehicles to spread lipid peroxides considered here include plasma membrane-derived micellar or vesicular structures, or potentially lysosomal vesicles or exosomes, which could additionally carry iron-loaded ferritin (Figure 2). It’s important to point out for this working model that lipid peroxides and iron are considered as spreadable factors, but distinguishing their cell-autonomous activities in mediating ferroptotic death from *in trans* activities may not be trivial.

Long-range spreading over large distances may require sensitization of cells to incoming signals, achieved experimentally by depletion of glutathione, and potentially facilitated *in vivo* through lowered antioxidant capacity, changes in iron content, or possibly alterations in lipid composition that could define the populations meant to be propagative (e.g. high levels of PUFAs) versus those that are not (low levels of PUFAs) (Figure 1)^[13,73]^. In this manner, propagation could be restricted to the appropriate cell types that need to be cleared, for example, to achieve the developmental sculpting of tissues. While ferroptosis propagation has been shown to play a role in muscle sculpting, other cell populations in the developing avian limb including ectoderm were also shown to exhibit hallmarks of ferroptosis^[13]^, and many other cell types and structures are more generally known to be remodeled, or even completely removed, to facilitate organismal development^[74]^. It is possible, then, that other propagative deaths could help to configure complex tissues, as may be observed, for example, during the pruning of epithelium from adult wings in fly development that has been shown to be propagative^[75]^. While death propagation may emerge with expanding roles in normal physiology, this property might also be useful as a therapeutic approach to treat cancer^[28,76]^. We note that ferroptosis propagation initiated in cancer cells could be highly propagative due to the starved nature of cancerous tissues, which often lack sufficient vascularization to deliver essential nutrients such as amino acids. In this case, ferroptosis initiated in cancer cells could spread and even eliminate non-cancer cells that are also nutrient-deprived within the local microenvironment. While the effect of death propagation on therapeutic responses is at present unknown, the observed modulation of ferroptosis sensitivity of xenograft tumors through loss or gain-of-function approaches for Gal13 suggest that ferroptosis propagation may occur and affect treatment responses *in vivo*^[60]^.

In consideration of a working model to explain ferroptosis propagation, here we highlighted potential lipid sources spreading from the plasma membrane or lysosomes. Lysosomes may be particularly interesting because they become multivesicular and could thereby release vesicles upon fusion to the plasma membrane. But many other intracellular membranes could also play a role, as peroxidation can also occur on mitochondria, the endoplasmic reticulum, and lipid droplets^[77–79]^, and additional endomembrane sources such as Golgi vesicles or early endosomes could also participate^[80,81]^. Along these lines, while we have used the term “lipid peroxide” broadly, whether particular sets of lipid species may be the most ideal propagative vehicles should also be considered^[82]^. It is also possible that products generated downstream of lipid peroxidation, such as fatty acid derivatives removed from phospholipids, or toxic aldehyde products (e.g., 4-Hydroxy-2-nonenal (4-HNE)), could be diffusible factors that could also propagate death. Finally, while we have considered models for the propagation of ferroptosis, it is possible that other types of regulated death might also share this unusual non-cell-autonomous feature, and thus further exploration of additional regulated mechanisms could contribute to defining a broader working model for death propagation more generally. To this point, the death mechanism pyroptosis was recently shown to have propagative activity, in this case mediated by the spreading of plasma membranes pores formed by gasdermin D from the plasma membrane of one cell to another, through extracellular vesicles formed by ESCRT-mediated membrane repair^[83]^, a mechanism reminiscent of models discussed here. Future approaches merging mechanistic cell biology with the study of physiology and disease, and potentially incorporating research from different forms of cell death, may reveal new insights into whether the minimal models discussed here can possibly explain this unusual phenomenon.

## Data Availability

Not applicable.
